# The effect of TEE on treatment change in patients with acute ischemic stroke

**DOI:** 10.1371/journal.pone.0243142

**Published:** 2020-12-03

**Authors:** Polina Specktor, Sergey Yalonetsky, Yoram Agmon, Elliot Sprecher, Faten Haj Ali, Gregory Telman

**Affiliations:** 1 Department of Neurology, Rambam Health Care Campus, Haifa, Israel; 2 Department of Neurology, Carmel Hospital, Haifa, Israel; 3 Technion, Israel Institute of Technology, Haifa, Israel; Jagiellonian University Medical College, POLAND

## Abstract

**Background and purpose:**

Ischemic stroke is a widespread disease carrying high morbidity and mortality. Transesophageal echocardiography (TEE) is considered an important tool in the work-up of patients with acute ischemic stroke (AIS) and transient ischemic attack (TIA) patients; its utility is limited by a semi-invasive nature. The purpose of this study was to evaluate the probability of treatment change due to TEE findings (yield) in the work-up of AIS and TIA patients.

**Methods:**

Retrospective data on patients with AIS or TIA who underwent TEE examination between 2000–2013 were collected from the institutional registry.

**Results:**

The average age of 1284 patients who were included in the study was 57±10.4, 66% of patients were male. The most frequent TEE findings included aortic plaques in 54% and patent foramen ovale (PFO) in 15%. TEE findings led to treatment change in 135 (10.5%) patients; anticoagulant treatment was initiated in 110 of them (81%). Most common etiology for switch to anticoagulation was aortic plaques (71 patients); PFO was second most common reason (26 patients). Significant TEE findings (thrombus, endocarditis, tumor) were found in 1.9% of patients, they were more common in young patients (<55; 56% of the patients).

**Conclusions:**

The beginning of anticoagulation treatment in patients with thick and complicated plaques was found frequently in our study. Significant TEE findings, were infrequent, constituted an absolute indication for treatment change and were more common in younger patients.

## Introduction

Ischemic stroke is one of the most prevalent diseases in the western world and is a second cause of mortality after acute coronary disease. One of six adults will suffer acute ischemic stroke (AIS) in his or her life [1]. In Israel, the annual incidence of stroke is approximately 18,500 patients per year [2]. Up to 30% of ischemic strokes is due to embolic etiology [3, 4]. Aside from the most common cause for cardiac embolic stroke, which is atrial fibrillation, other common etiologies include aortic plaques and patent foramen ovale (PFO).

Transesophageal echocardiography (TEE) is considered an important tool in the work-up of patients with AIS [5–7]. Several studies have compared the efficacy of TEE with non-invasive transthoracic echocardiography [TTE] in AIS patients. While some researchers doubt the advantage of TEE over TTE [8, 9], most studies confirm the superiority of TEE in the detection of cardiac thrombi, PFO, atrial septal defect (ASD) and aortic plaques [10–14]. The risk of ischemic stroke changes according to the cardiac abnormality (for example, patients with an artificial valve have a greater risk for stroke than patients with PFO); many patients have two or more coexistent cardiac pathologies [4, 15].

As TEE is semi-invasive and relatively expensive [16], it is essential to determine the probability of treatment change due do TEE findings (yield), which ranges in the literature from 0% to 11% [8, 12, 13, 17–20]. Several studies further tried to find subpopulations with higher TEE yield. Some support routine TEE use in younger patients [21, 22], yet others in an elderly stroke population [20].

The aim of this study was to evaluate the yield of TEE in northern Israel AIS and transient ischemic attack (TIA) populations and examine whether it differs with age.

## Materials and methods

The study is a retrospective study, based on data from computerized system used at Rambam Health Care Campus in Haifa, Israel. It was reviewed and approved by Institutional Review Boards of Rambam Health Care Campus (# 3023 IRB). The data was fully anonymized before accession to patients’ data including demographic and risk factors, clinical status, work-up results, treatment regimens, and discharge details. Written informed consent from the participants was not required to participate in this study in accordance with the national legislation and the institutional requirements.

TEE is routinely performed in AIS and TIA patients, hospitalized in Rambam’s Neurological department, answering the following criteria: (1) patients 55 years old and younger (2) patients above 55 with recurrent strokes or with suspected embolic etiology (clinically or radiologically). All TEE studies were performed by senior cardiologists with a subspecialty in echocardiography, including TEE. Examinations were performed on the Acuson Sequoia 512 machine (Siemens Medical Solutions, USA).

Data on 1284 patients with AIS or TIA who underwent TEE as part of the work-up during hospitalization in the Department of Neurology from 1.1.2000 to 31.12.2013 were used in the study. This study includes all stroke and TIA patients who underwent TEE during the study period.

The assignment of ethnicity (Arab or Jewish) was determined by place of birth and residence in addition to first and family names. A preliminary inter-observer agreement study showed almost perfect agreement.

Stroke was defined as an acute neurologic deficit of vascular cause persisting for more than 24 hours after symptom onset with no evidence of brain hemorrhage or other cause, while TIA was defined as a neurologic deficit of presumed vascular cause that resolved within the first 24 hours after symptom onset.

Ischemic stroke location was detected based on the imaging results (brain computed tomography and/or magnetic resonance imaging) or clinical evaluation, if the imaging did not provide conclusive results.

Hypertension was defined by a prehospital diagnosis of hypertension or by the use of antihypertensive medication. Cases that did not have a prior diagnosis of hypertension were diagnosed based on repeated measurements of blood pressure (and its values ≥140/90) through the hospitalization. Diabetes mellitus was defined by a prehospital diagnosis of diabetes mellitus; recording of random blood glucose levels ≥200 mg/dL or by the use of intravenous or oral hypoglycemic agent. Hyperlipidemia was defined by a prehospital diagnosis of hyperlipidemia; prior use of lipid-lowering medications or by blood cholesterol and lipid values (fasting serum total cholesterol concentration of >200 mg/dL; low-density lipoprotein cholesterol concentration of >100 mg/dL; high-density lipoprotein cholesterol concentration of <40 mg/dL; triglyceride concentration of >150 mg/dL). Atrial fibrillation was diagnosed based on previous medical records or by a physician who reviewed the patients’ electrocardiograms or Holter recording. Ischemic heart disease (IHD) was defined by a previous record of IHD, a history of myocardial infarction or angina pectoris, or signs of old ischemia on the ECG recording. Peripheral vascular disease (PVD) was based on a prehospital diagnosis or a history of intermittent claudication, peripheral vascular surgery, or angioplasty.

TEE was performed during hospitalization by a single Board-certified cardiologist according to standard practice guidelines, as previously described.

In cases with high suspicion of cardioembolic event, TEE was performed in 24 hours after admission. High suspicion of cardioembolic stroke was established in cases with several strokes in different vascular territories presented clinically or by neuroimaging simultaneously or with temporal distribution.

Most patients with known AF and large artery or lacunar stroke were excluded from the study as TEE did not have added value in change of treatment in those patients. Patients diagnosed with AF during hospitalization before or after TEE performance and cases when cardiologist

recommended TEE in spite of known AF were included in the study.

The thoracic aorta (ascending aorta, aortic arch, and descending thoracic aorta) was imaged in long- and short-axis views. The presence of left atrial and left ventricular thrombi, PFO, aortic atheroma, thrombus and tumor were recorded. In addition, the presence of LV dysfunction, LV hypertrophy and regional LV wall motion abnormalities were noted.

Aortic atherosclerotic plaques were defined as focal structures encroaching into the arterial lumen. Small plaques were defined as 4 mm thick and less, while large plaques included plaques thicker than 4 mm. Complex plaques were defined as ulcerated plaques (by visual assessment) or plaques with mobile debris, regardless of their size.

Small PFOs were defined as PFO below 2 mm, moderate between 2 and 4 mm and large PFOs were PFOs larger than 4 mm. Existence of shunt through PFO lumen was evaluated at rest and during Valsalva maneuver by color Doppler.

Yield of TEE was defined as change of medical treatment or procedure performed based on TEE findings.

The distribution of demographic data, risk factors and abnormalities shown on TEE was calculated by probability index, standard deviation and standard error. The yield of TEE was calculated by percentage of change of treatment or procedure due to TEE findings.

Distributions of reported continuous parameters were examined and summarized appropriately, and subgroups of nominal and ordinal parameters were tabulated. For analysis purposes, age was converted to three subgroups. Cross-tabulations of potential predictors of treatment change were assessed using Likelihood ratio chi-square. Statistical analysis was performed using JMP (SAS Institute, Cary, NC).

## Results

A total of 1284 patients who consecutively underwent TEE were included in the study. The mean age of the population included in the study was 57±10.4 years. Distribution of demographic parameters, vascular risk factors and vascular disease is presented in Table 1.

**Table 1:** Distribution of demographic parameters, vascular risk factors and vascular diseases in the whole group of patients.

Demographic data (n = 1284)	
Age (years±SD)	57±10.4
Gender (males)	855 (66%)
Ethnicity (Jews)	953 (74%)
Vascular risk factors and diseases	
Hypertension	841 (65%)
Diabetes	454 (35%)
Smoking	415 (32%)
Hyperlipidemia	706 (55%)
Atrial fibrillation	48 (4%)
Ischemic heart disease	277 (21%)
Peripheral vascular disease	42 (3%)

Atrial fibrillation (AF) was recorded in 4% of patients; main indication for performance of TEE in those patients was high index of suspicion of left atrial thrombus.

Eighty-seven percent of the patients suffered from AIS, the rest were TIA patients. Seventy-three percent of the clinical events took place in the carotid territory; 489 (38%) of the strokes and TIAs were in the left carotid territory, 460 (35%) of the clinical syndromes were in the right carotid territory, 278 (22%) were in the vertebro-basilar territory, and in 60 cases the territory of the infarct was not definitely identified.

**Fig 1:**
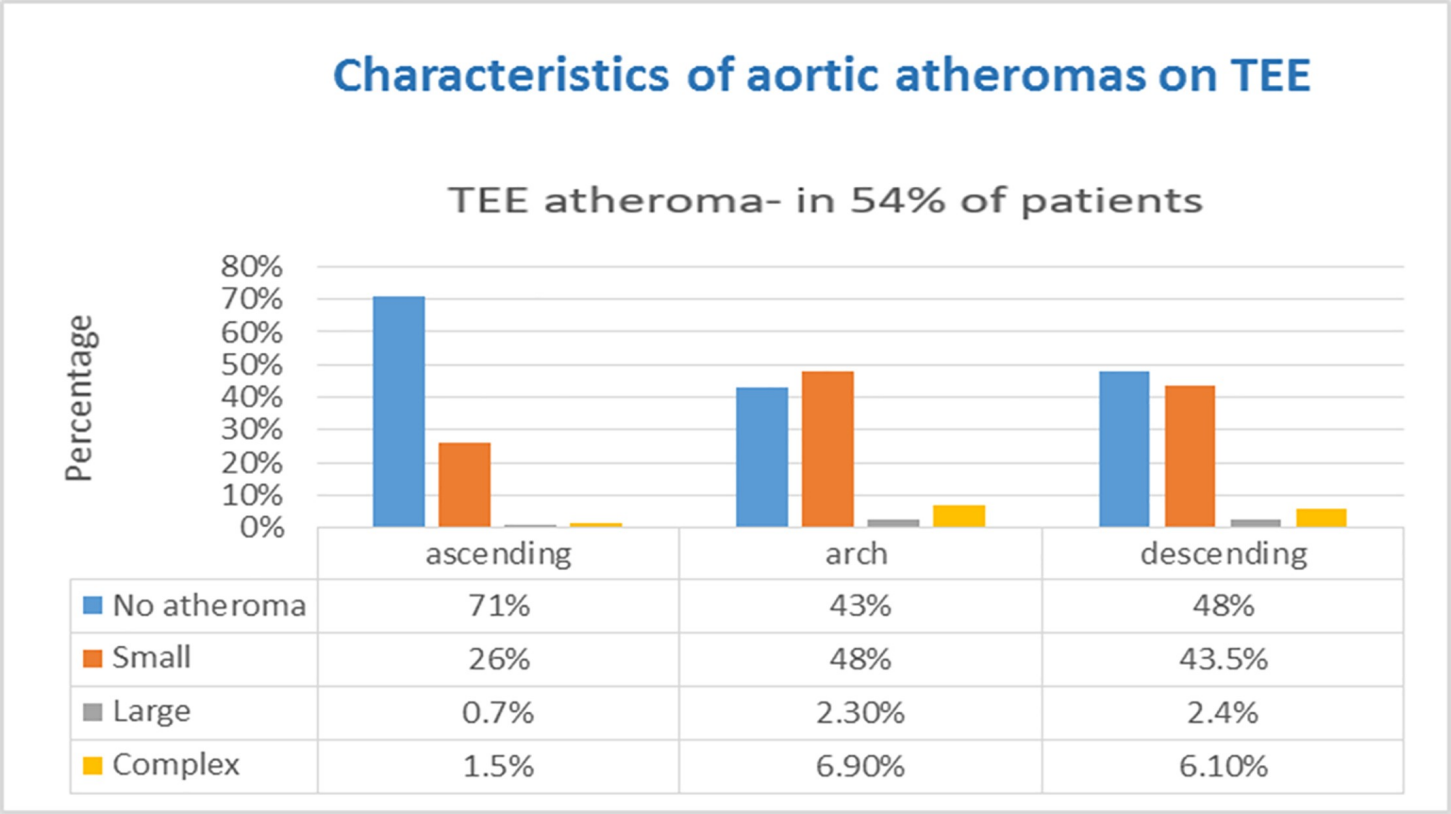
Characteristics of aortic atheromas. TEE- transesophageal echocardiography; Small atheroma- 4 mm thick plaques and less; Large atheroma- plaques thicker than 4 mm; Complex atheroma- plaques that are ulcerated or with mobile debris, regardless of their size.

TEE findings are presented in Table 2. Six hundred ninety-seven (54%) patients had any aortic plaques; PFO was the second most frequent finding, detected in 199 (15%) patients. PFOs were further classified according to their size and presence of right to left shunt (Table 2). Aortic plaques were classified further by size and location (Fig 1).

**Table 2:** TEE findings in stroke and TIA patients.

Aortic atheroma	697 (54%)
PFO	199 (15%)
Small	135 (10.5%)
Moderate	32 (2.5%)
Large	32 (2.5%)
Presence of shunt	132 (10%)
Endocarditis	8 (0.6%)
Tumor	4 (0.3%)
Severe systolic dysfunction	36 (2.8%)
Thrombus	13 (1%)
Severe valve defect*	20 (1.5%)

* Mitral or aortic regurgitation/ stenosis considered severe upon TEE imaging.

Change of treatment due to TEE findings was made in 135 patients; patients with AF were excluded from the analysis. Overall the yield of TEE in our study was 10.5%. Anticoagulation instead of antiplatelet therapy was the most frequent change (110 patients, 81.5%), see Table 3. Complex aortic plaques and thick plaques more than 4mm in the arch were the main reason for treatment change (66 patients, 49%). Second most common reason was PFO (26 patients). The whole distribution of etiologies leading to the change of treatment is presented in Table 4.

**Table 3:** Treatment change in the whole group of patients due to TEE findings.

Treatment/procedure	Patients (%)
Anticoagulants	110 (8.5%)
Closure of PFO	13 (1%)
Antibiotics	4 (0.3%)
Closure of PFO + Anticoagulants	4 (0.3%)
Surgery	4 (0.3%)

**Table 4:** The most significant TEE finding responsible for treatment change, according to age groups (Likelihood ratio chi-square: P value = 0.0001).

Age	<55	55–65	˃65	Total
Number of patients (total)	471	547	266	1284
Severe heart failure	3 (2%)	7 (5%)	3 (2%)	13 (10%)
Plaques*	10 (7%)	39 (29%)	22 (16%)	71 (53%)
PFO**	14 (10%)	10 (7%)	2 (1.5%)	26 (19%)
Absolute indication***	14 (10%)	5 (3%)	6 (4%)	25 (18.5%)
Total	41 (30%)	61 (45%)	33 (24.5%)	135

*Fifty three of the plaques were complex, thirteen were large plaques, and the rest were small plaques.

**All cases but one had PFO with shunt.

***tumor, thrombus or endocarditis.

Definite indications for treatment change, according to 2014 AHA guidelines, include cardiac thrombus (13 patients, 9.63%), endocarditis (8 patients, 5.92%) and cardiac tumor visualized in 4 (2.96%) patients. Thus, only 1.9% of our study population had a definite indication for treatment change (level of evidence A) following TEE. Significant TEE findings were more common in young patients (<55; 56% of the patients). In other patients, treatment change was made due to relative indications (level of evidence C, [23]).

**Fig 2:**
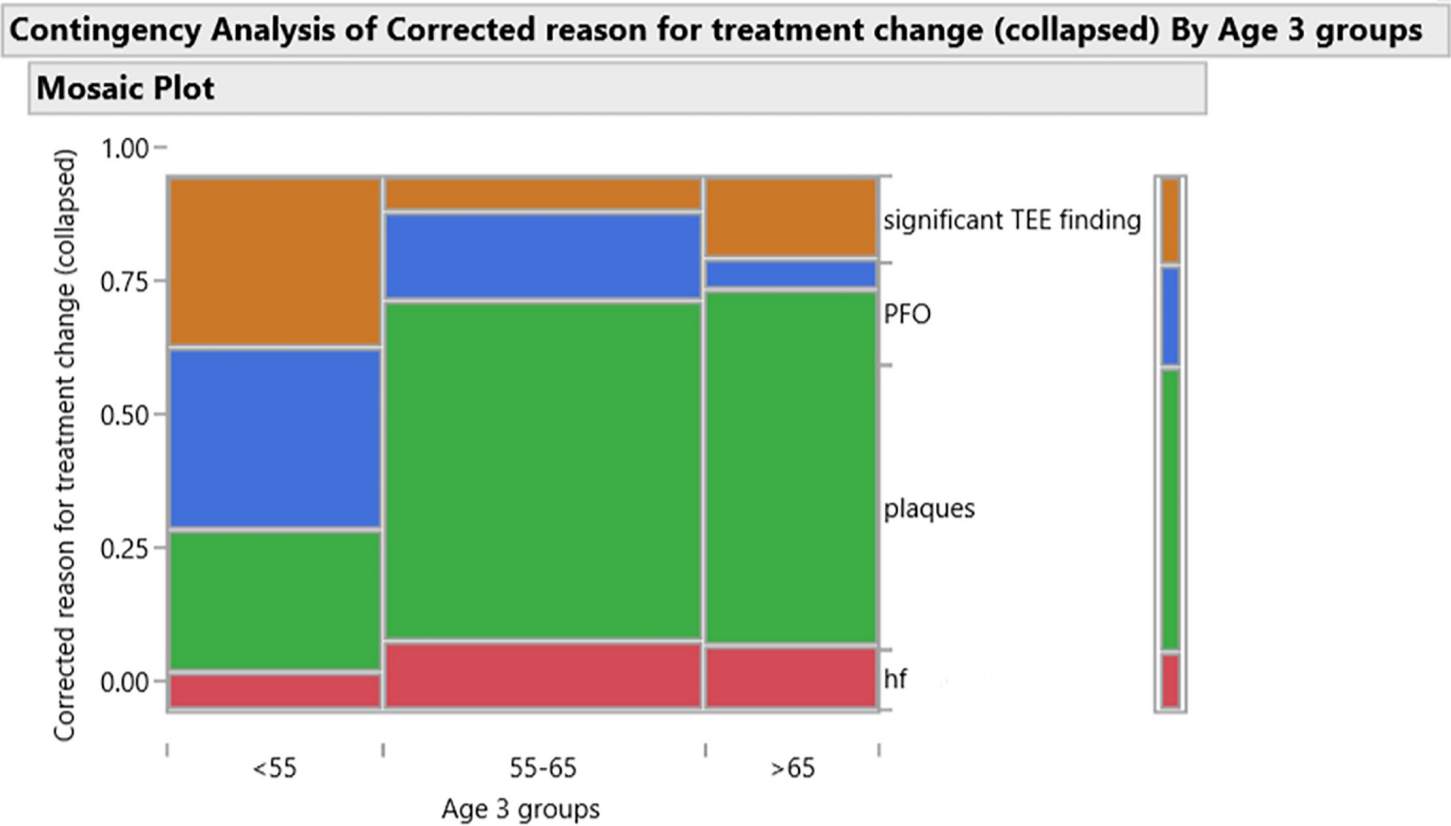
Contingency analysis of corrected reason for treatment change (collapsed) by age groups. Significant TEE finding- tumor, thrombus or endocarditis; PFO- patent foramen ovale (25 cases with shunt, one case without shunt); plaques- 53 complex, 13 large, 5 small; Hf- severe heart failure.

TEE yield was highest in patients ˃65 years old (12.4% vs. 10.5% in all age groups), treatment change following PFO and significant findings was highest in patients < 55 years old (5.9% vs 4% in all age groups), Table 4, Fig 2.

**Fig 3:**
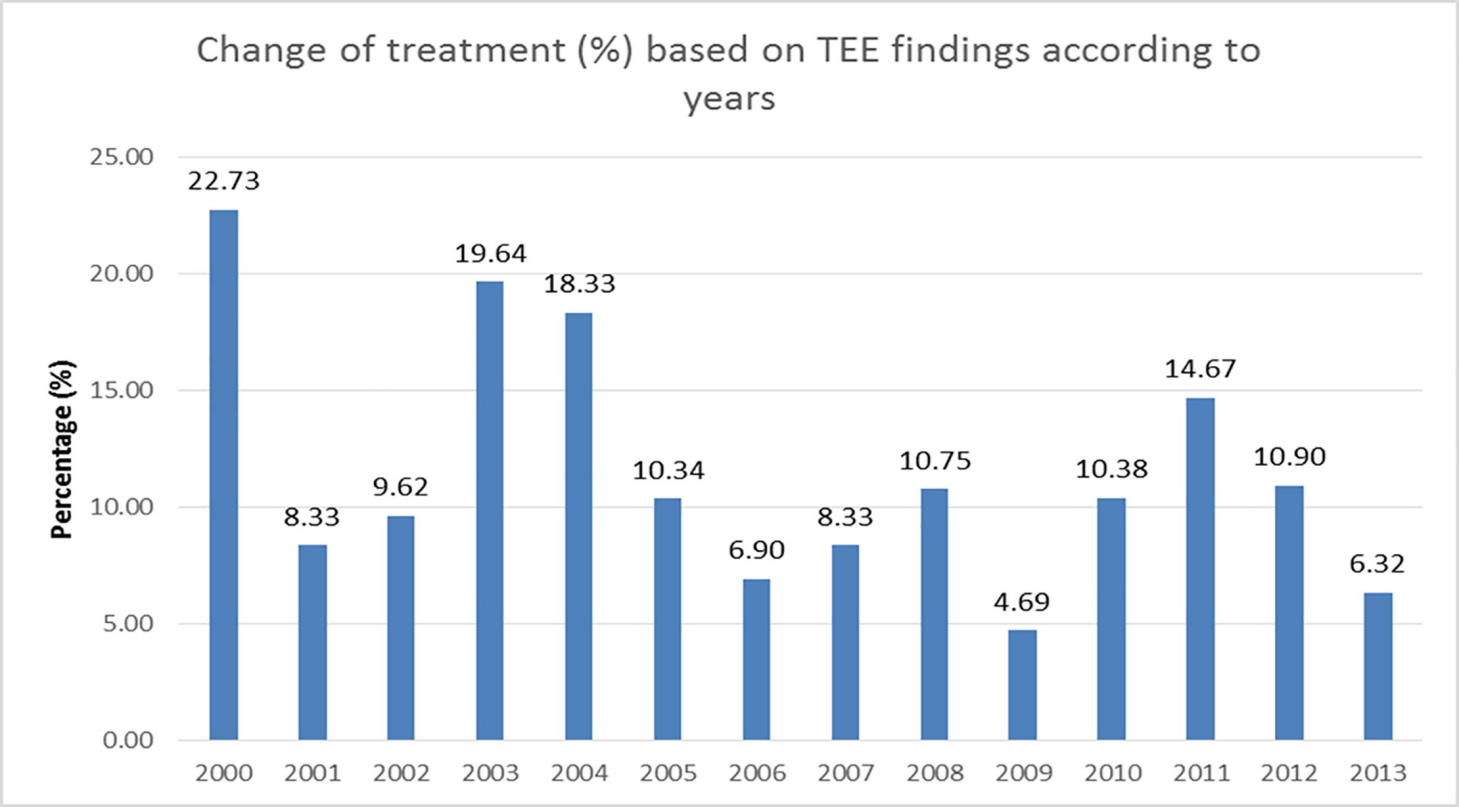
Temporal distribution of treatment changes due to TEE findings in patients who underwent TEE (descriptive statistics, no statistical significance). TEE- transesophageal echocardiography.

Temporal analysis of treatment change through the years showed large fluctuations, with a maximum of 22.7% of treatment change in 2000 and a minimum of 4.7% in 2009 (Fig 3).

## Discussion

We found a relatively high yield of TEE during the study period from 2000 to 2013. Our data showed that the yield of TEE changes according to changing indications for treatment. Through the years many of the “classic” indications (PFO, atheromas, heart failure) became “relative” ones; in the recent years, there has been a tendency for a reduction of the yield of TEE.

Among the “relative” indications, warfarin treatment for thick and complex aortic plaques was the most frequent. The risk for recurrent stroke for patients with aortic plaques thicker than 4 cm is significantly greater than in patients without aortic plaques [24]. The risk is twelve times higher if the plaques are ulcerated [25]. Protruding atheromas of the ascending aortic arch are best diagnosed by TEE [26]. According to the literature, they can be found in 10–20% of patients undergoing TEE after ischemic stroke [10, 27, 28]. These data match our results. Although the relationship between large and complicated aortic plaques and AIS is generally accepted, the optimal treatment (anticoagulation versus antiplatelet therapy) is not definite [29–31].

The frequency of PFO in our study correlates with previous studies [32]. PFO is associated with ischemic strokes and TIAs by mechanism of paradoxical embolus [33]. This relationship is probably stronger in younger adults with cryptogenic stroke [34, 35]. PFO is better visualized by TEE than by TTE [36]. At the present time, the optimal treatment for stroke patients with PFO is unsettled. Most experts suggest antiplatelet therapy for these patients as a first line of treatment [29, 37]. Patients with PFO and a proven source of thromboembolism (DVT, for example, proven by leg Doppler sonography) may receive anticoagulant treatment for three months [29]. Until recently, PFO closure was not considered a risk reducing procedure for recurrent stroke [29, 38–40]. New trials in 2017–2018 questioned this policy and suggested considering PFO closure in addition to lifelong antiplatelet treatment in selected patients [41, 42]. These results may again raise the yield of TEE in upcoming years.

Treatment change according to absolute indications (thrombus in the left atrium or ventricle, endocarditis and cardiac tumor) was performed in a small part of the study population.

Cardiac thrombus is a rare cause for ischemic stroke; its prevalence is 0.1% in patients with sinus rhythm and without stroke [9] and rises to 1% in patients with stroke or TIA [43]. Our study showed a low prevalence of thrombus in AIS patients as well. We found that more than half the patients with thrombus did not have a prior history or TEE findings of significant heart disease. Thrombus in the left ventricle is adequately diagnosed by both TEE and TTE, but TEE seems to be more sensitive for the diagnosis of thrombus in the left atrium [9]. There is a consensus that this is a definite indication for anticoagulation [29].

Endocarditis, both infective and nonbacterial, is a well-documented cause of ischemic stroke. In fact, ischemic stroke is the most prevalent complication of infective endocarditis (IE), occurring in about 35% of cases [44]. The most sensitive imaging modality for IE is TEE [45–47]. As endocarditis has a high mortality and morbidity [48, 49], it requires prompt diagnosis and treatment. The incidence of endocarditis in our study was less than 1%, whereas previous studies in younger patients showed the rate of nonbacterial and infectious endocarditis in stroke patients as high as 4–6% [50–52]. Plausible explanation is the younger age of endocarditis patients, we witnessed in our study.

Our study showed a low incidence of cardiac tumor in patients with ischemic stroke. Although the incidence of cardiac tumor reported in non-stroke population is 1:1000, our number is compatible with previous studies showing a higher incidence of cardiac tumor in patients with ischemic stroke [4, 53]. TEE seems to be superior than TTE in the diagnosis of cardiac tumors [54, 55].

The study showed that TEE findings vary with age, influencing TEE yield. Absolute indications for treatment change (tumor, thrombus and endocarditis) as well as PFO were most frequent <55 years, suggesting a higher yield in this population. This finding correlates with previous studies suggesting a higher TEE yield in younger patients [21, 22, 56].

Our study has several limitations. First, we did not gather baseline data regarding previous IS or TIA. Second, AIS patients who had TEE were not consecutive and thus selection bias exists. Third, this is retrospective analysis; data were collected mainly from an electronic system which may lead to incomplete documentation in some cases. However, we believe that the large number of patients included in our study provides the possibility of obtaining a real world picture and developing reliable conclusions about the role of TEE in the work-up of AIS patients.

## Conclusions

Overall, we found a higher TEE yield in older patients. However, considering changing guidelines, in the recent years TEE yield is higher in younger patients (<55). The neurologist should consider a TEE examination in the AIS and TIA populations according to the stroke pattern and preexisting conditions.

## Supporting information

S1 Data (XLSX)
